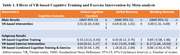# Virtual Reality‐based Cognitive and Exercise Intervention for People with Mild Cognitive Impairment. A systematic review and meta‐analysis

**DOI:** 10.1002/alz.085515

**Published:** 2025-01-09

**Authors:** Joyce YC Chan, Kelvin KF Tsoi

**Affiliations:** ^1^ The Chinese University of Hong Kong, Hong Kong China; ^2^ JC School of Public Health and Primary Care, The Chinese University of Hong Kong, Hong Kong China

## Abstract

**Background:**

Cognitive training and exercise intervention are suggested for enhancing cognitive functions in people with mild cognitive impairment (MCI). The use of virtual reality (VR) has been applied in cognitive training and exercise intervention in recent decades. However, it is still unclear whether VR‐based intervention is a useful means to enhance cognitive functions in people with MCI. Therefore, the objective of this meta‐analysis was to examine the effect of VR‐based cognitive training and exercise intervention on cognitive functions in people with MCI.

**Method:**

A systematic literature search was conducted via OVID databases, including Pubmed, Medline, Embase, and APA PsychoInfo. Randomized controlled trials (RCT) recruited people with MCI and examined the effect of VR‐based cognitive training and/or exercise intervention were included. The outcomes were global cognitive function and memory. All outcomes were measured by using standard cognitive assessment tools. The effects were pooled by meta‐analysis and standardized mean difference (SMD) was used to calculate the combined results. Subgroup analyses were conducted according to the type of VR‐based intervention.

**Result:**

Sixteen RCTs involving 889 people with MCI were included. The mean age ranged from 67 to 87. The length of intervention ranged from 4 to 20 weeks. People with MCI who received VR‐based intervention showed better global cognitive function (SMD = 0.51, 95% CI = 0.20 to 0.83), verbal memory (SMD = 0.64, 95% CI = 0.26 to 1.01) and working memory (SMD = 3.00, 95% CI = 1.31 to 4.69) than the control group. In subgroup analysis, VR‐based combined cognitive training and exercise intervention showed a large and significant effect in enhancing global cognitive function and verbal memory, whereas VR‐based exercise intervention showed a large and significant effect in improving working memory.

**Conclusion:**

The present study demonstrated that VR‐based intervention can be able to enhance global cognitive function and memory in people with MCI. VR‐based combined cognitive training and exercise intervention appear to have a larger effect on cognitive functions. However, the number of studies in sub‐group analyses was small. Further research on the mode of VR‐based interventions is suggested.